# Supplementary figures and images for: A new approach to quantify angles and time of changes-of-direction during soccer matches
Source: PLoS One. 2021 May 17;16(5):e0251292. doi: 10.1371/journal.pone.0251292 (PMC8128237; doi:10.1371/journal.pone.0251292)

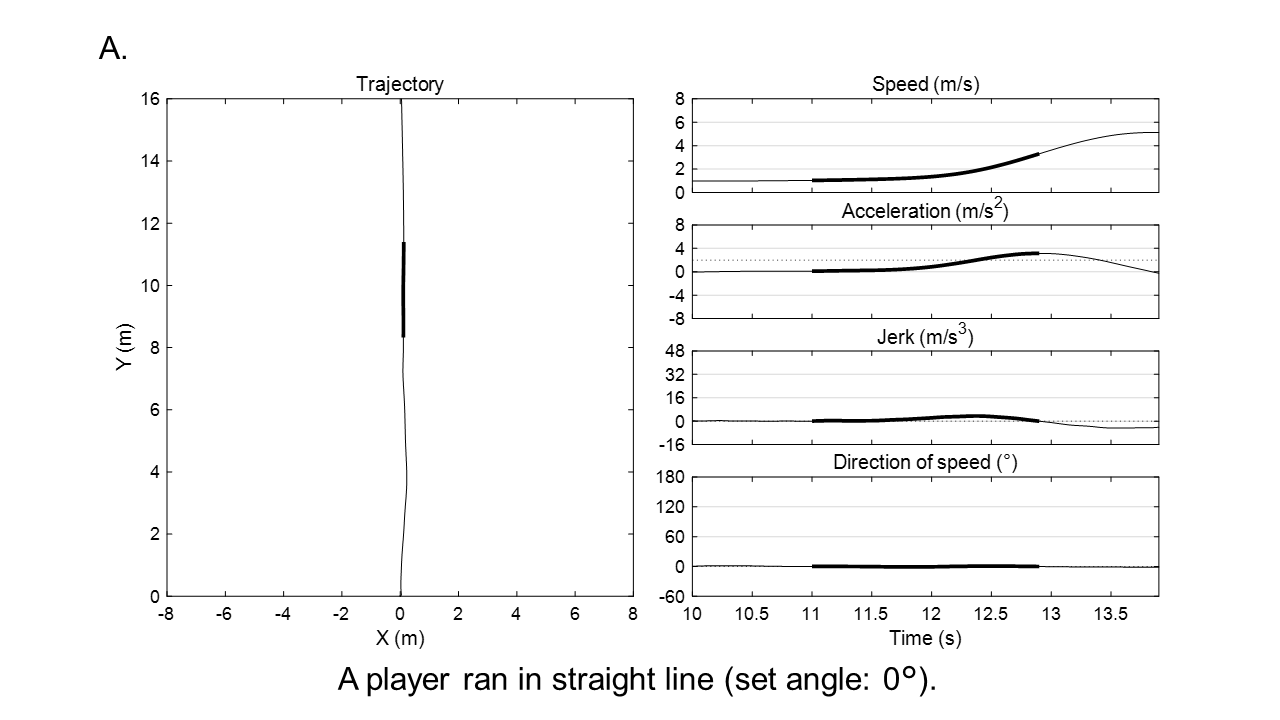

Supplement: S1 Fig — A: 0°, B: 15°, C: 30°, D: 45°, E: 60°, F: 75°, G: 90°, H: 105°, I: 120°, J: 135°, K: 150°, L: 165°, M: 180°. A bold line overlapped in line plot indicates an analytical period. (ZIP) [file pone.0251292.s001.zip › S1a_Fig.TIF]

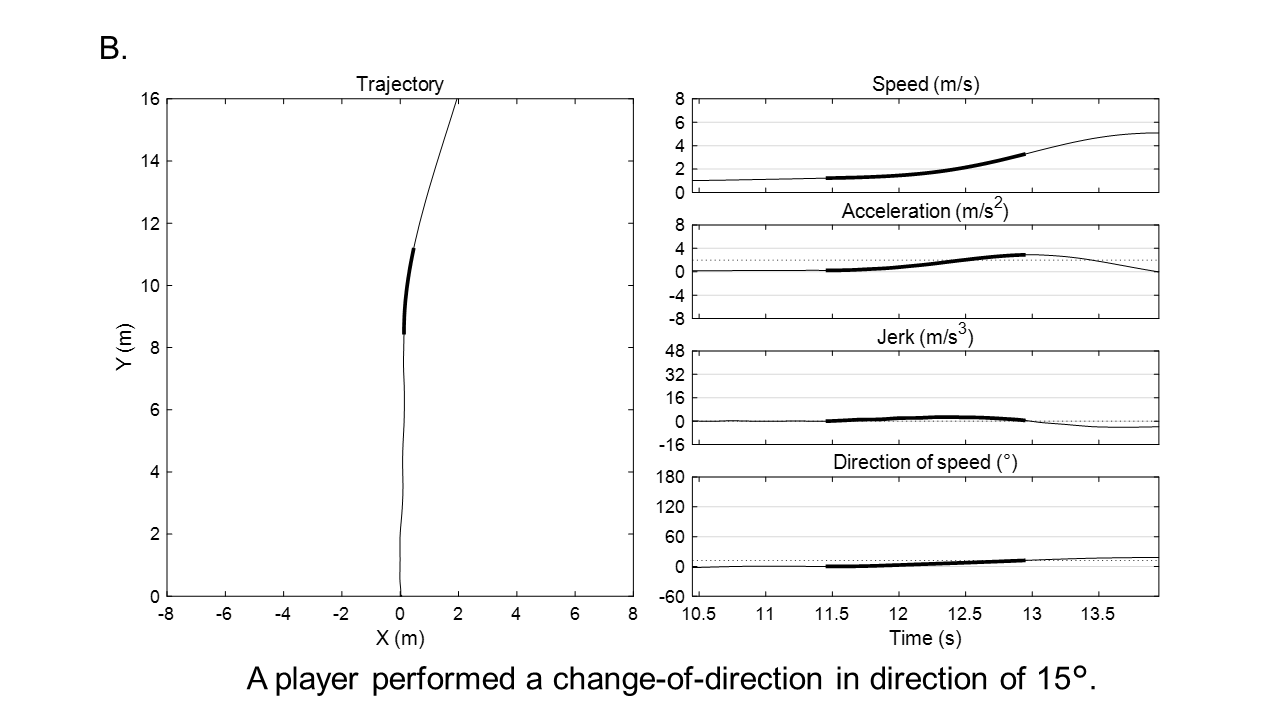

Supplement: S1 Fig — A: 0°, B: 15°, C: 30°, D: 45°, E: 60°, F: 75°, G: 90°, H: 105°, I: 120°, J: 135°, K: 150°, L: 165°, M: 180°. A bold line overlapped in line plot indicates an analytical period. (ZIP) [file pone.0251292.s001.zip › S1b_Fig.TIF]

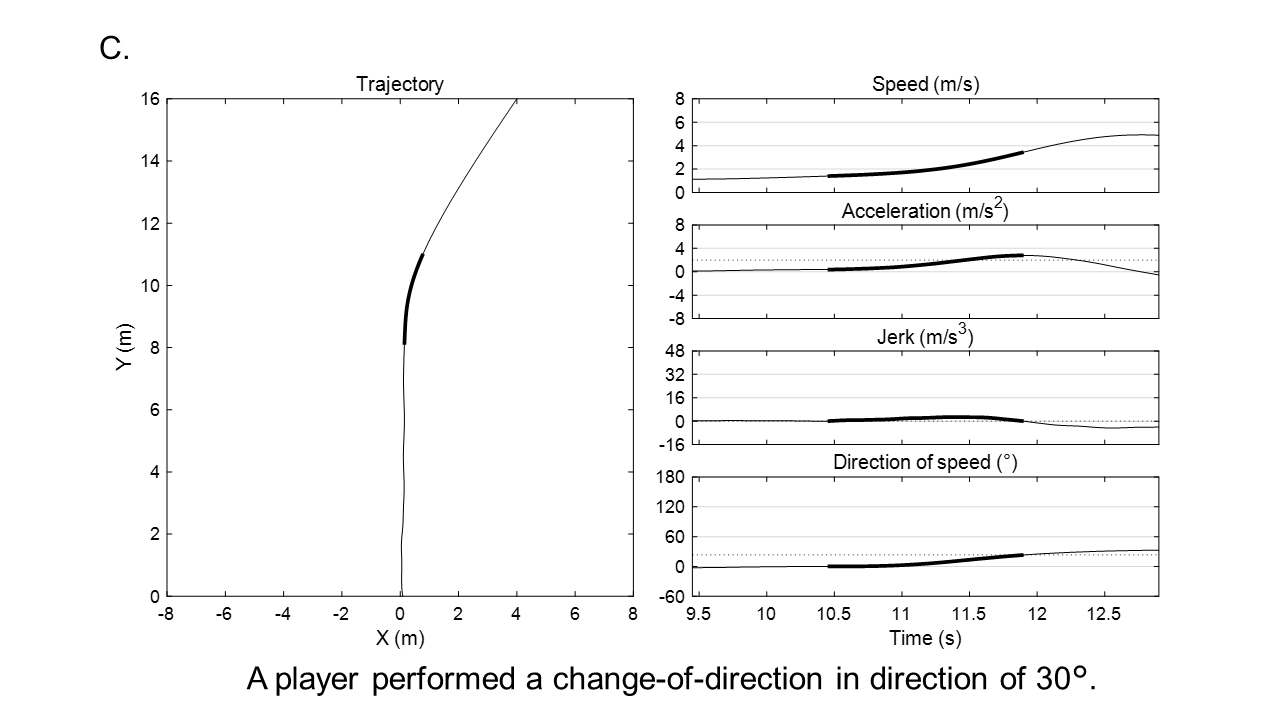

Supplement: S1 Fig — A: 0°, B: 15°, C: 30°, D: 45°, E: 60°, F: 75°, G: 90°, H: 105°, I: 120°, J: 135°, K: 150°, L: 165°, M: 180°. A bold line overlapped in line plot indicates an analytical period. (ZIP) [file pone.0251292.s001.zip › S1c_Fig.TIF]

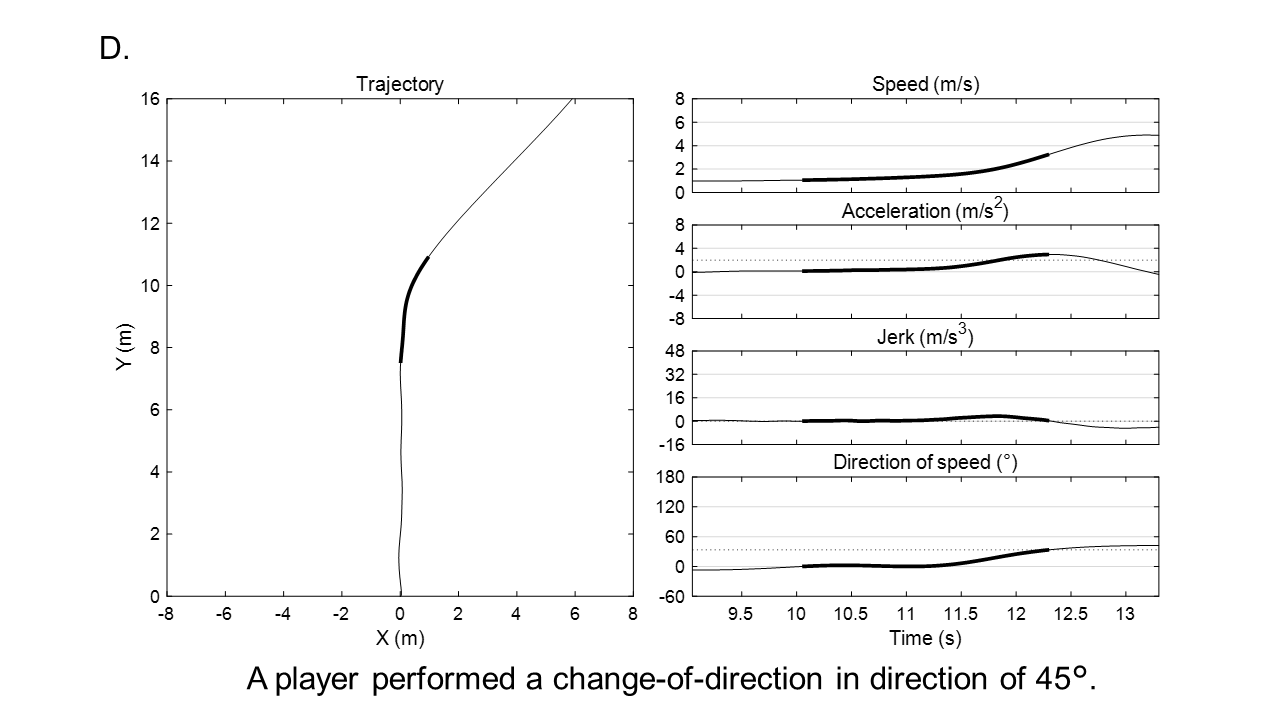

Supplement: S1 Fig — A: 0°, B: 15°, C: 30°, D: 45°, E: 60°, F: 75°, G: 90°, H: 105°, I: 120°, J: 135°, K: 150°, L: 165°, M: 180°. A bold line overlapped in line plot indicates an analytical period. (ZIP) [file pone.0251292.s001.zip › S1d_Fig.TIF]

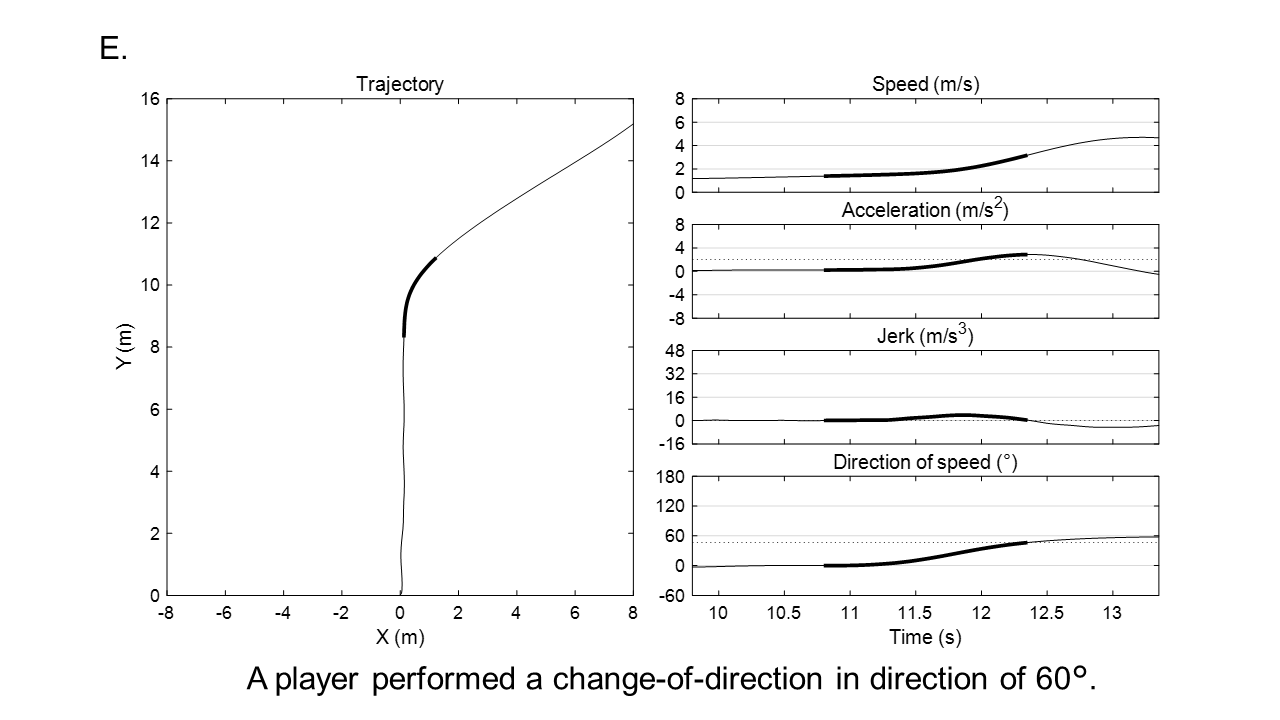

Supplement: S1 Fig — A: 0°, B: 15°, C: 30°, D: 45°, E: 60°, F: 75°, G: 90°, H: 105°, I: 120°, J: 135°, K: 150°, L: 165°, M: 180°. A bold line overlapped in line plot indicates an analytical period. (ZIP) [file pone.0251292.s001.zip › S1e_Fig.TIF]

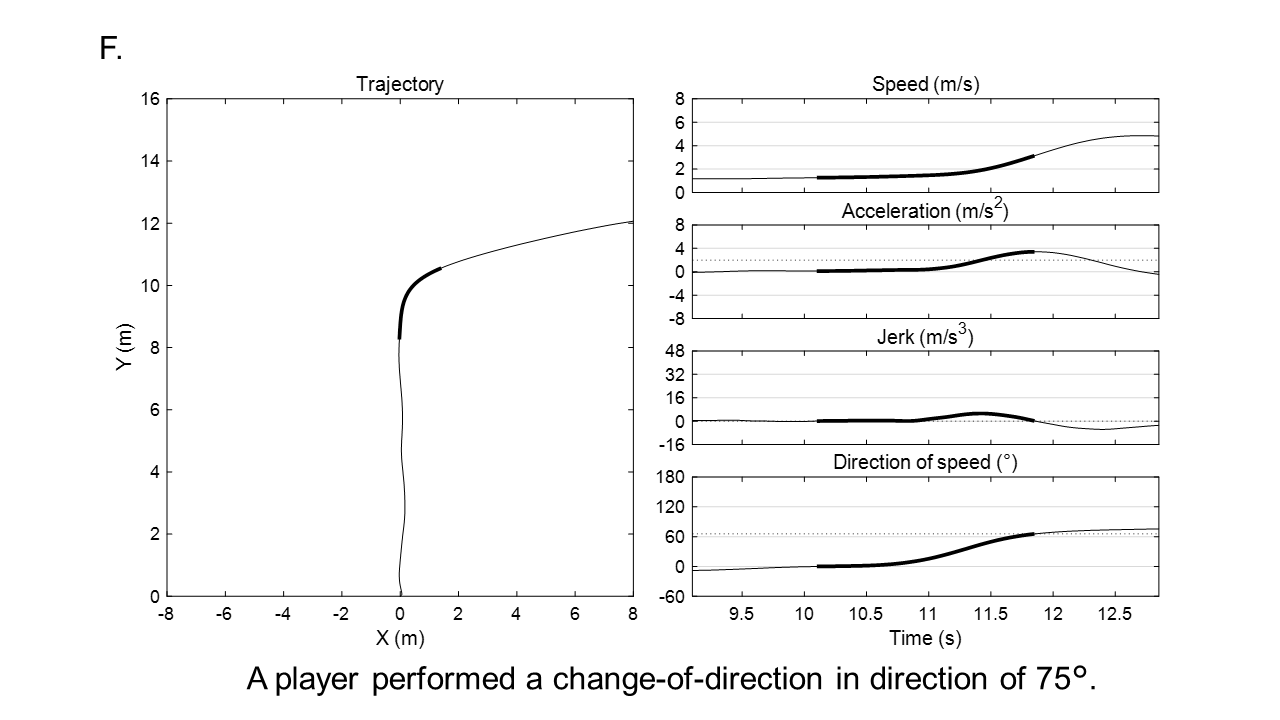

Supplement: S1 Fig — A: 0°, B: 15°, C: 30°, D: 45°, E: 60°, F: 75°, G: 90°, H: 105°, I: 120°, J: 135°, K: 150°, L: 165°, M: 180°. A bold line overlapped in line plot indicates an analytical period. (ZIP) [file pone.0251292.s001.zip › S1f_Fig.TIF]

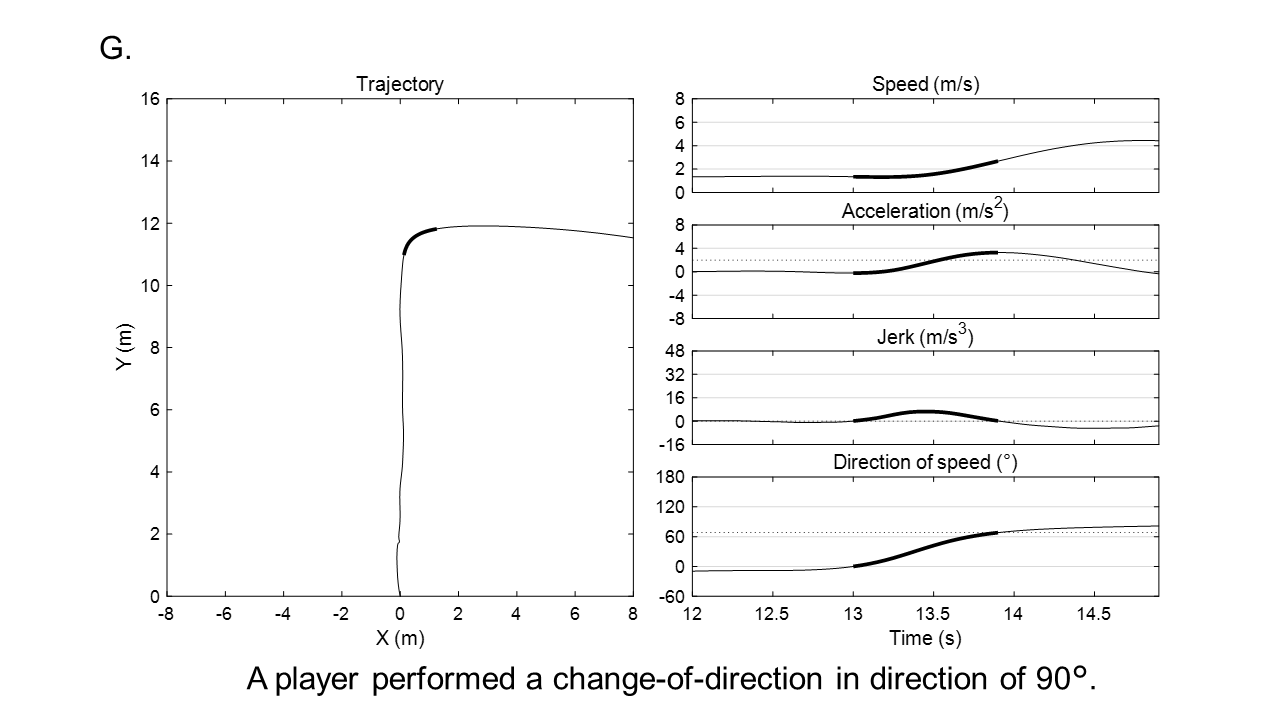

Supplement: S1 Fig — A: 0°, B: 15°, C: 30°, D: 45°, E: 60°, F: 75°, G: 90°, H: 105°, I: 120°, J: 135°, K: 150°, L: 165°, M: 180°. A bold line overlapped in line plot indicates an analytical period. (ZIP) [file pone.0251292.s001.zip › S1g_Fig.TIF]

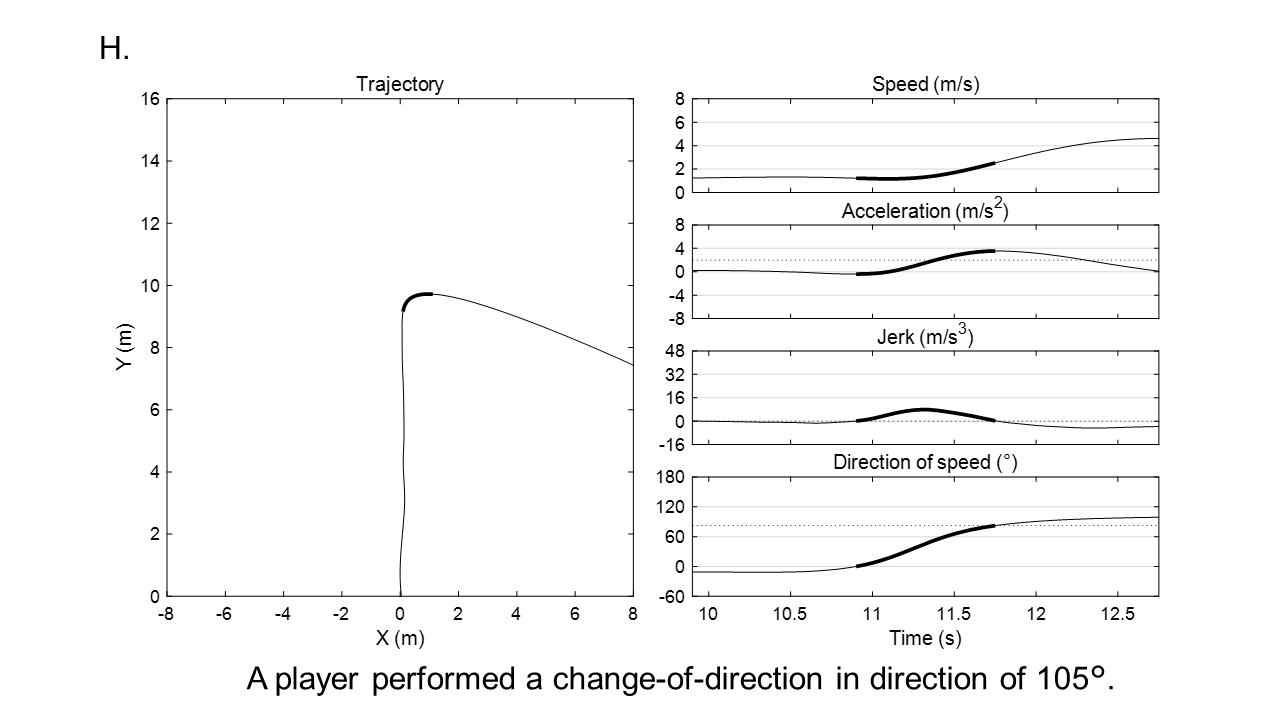

Supplement: S1 Fig — A: 0°, B: 15°, C: 30°, D: 45°, E: 60°, F: 75°, G: 90°, H: 105°, I: 120°, J: 135°, K: 150°, L: 165°, M: 180°. A bold line overlapped in line plot indicates an analytical period. (ZIP) [file pone.0251292.s001.zip › S1h_Fig.TIF]

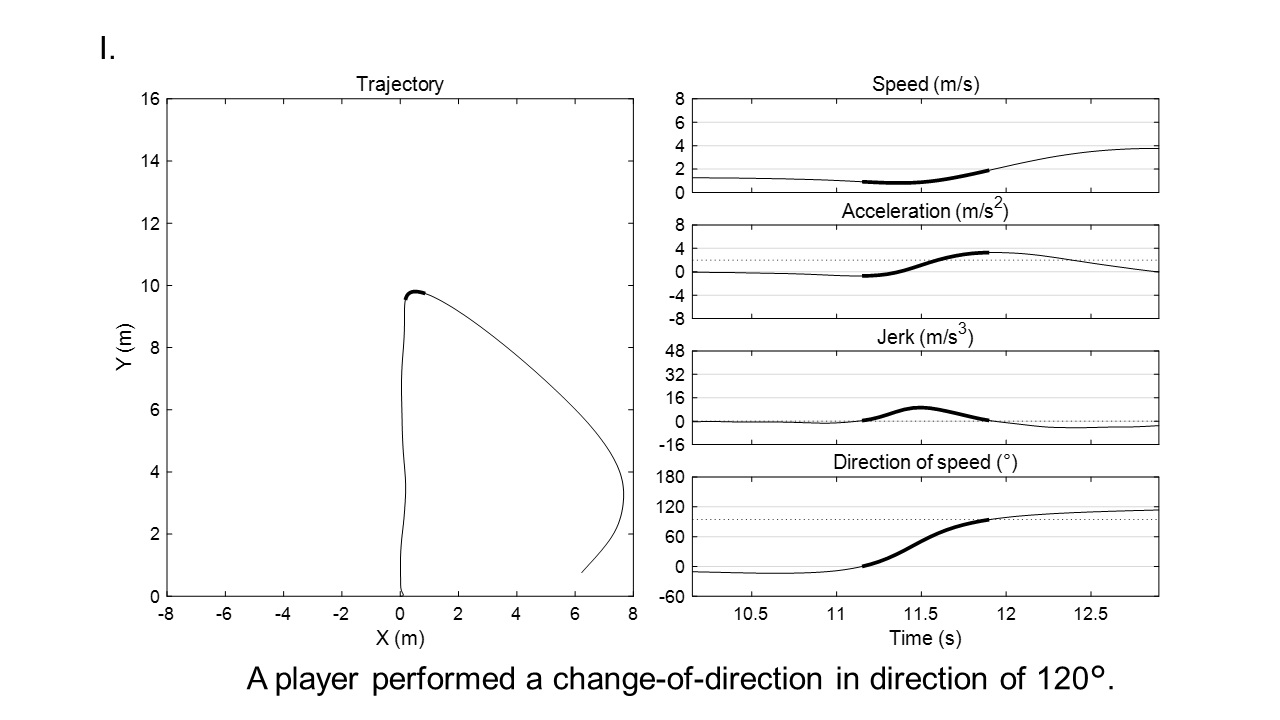

Supplement: S1 Fig — A: 0°, B: 15°, C: 30°, D: 45°, E: 60°, F: 75°, G: 90°, H: 105°, I: 120°, J: 135°, K: 150°, L: 165°, M: 180°. A bold line overlapped in line plot indicates an analytical period. (ZIP) [file pone.0251292.s001.zip › S1i_Fig.TIF]

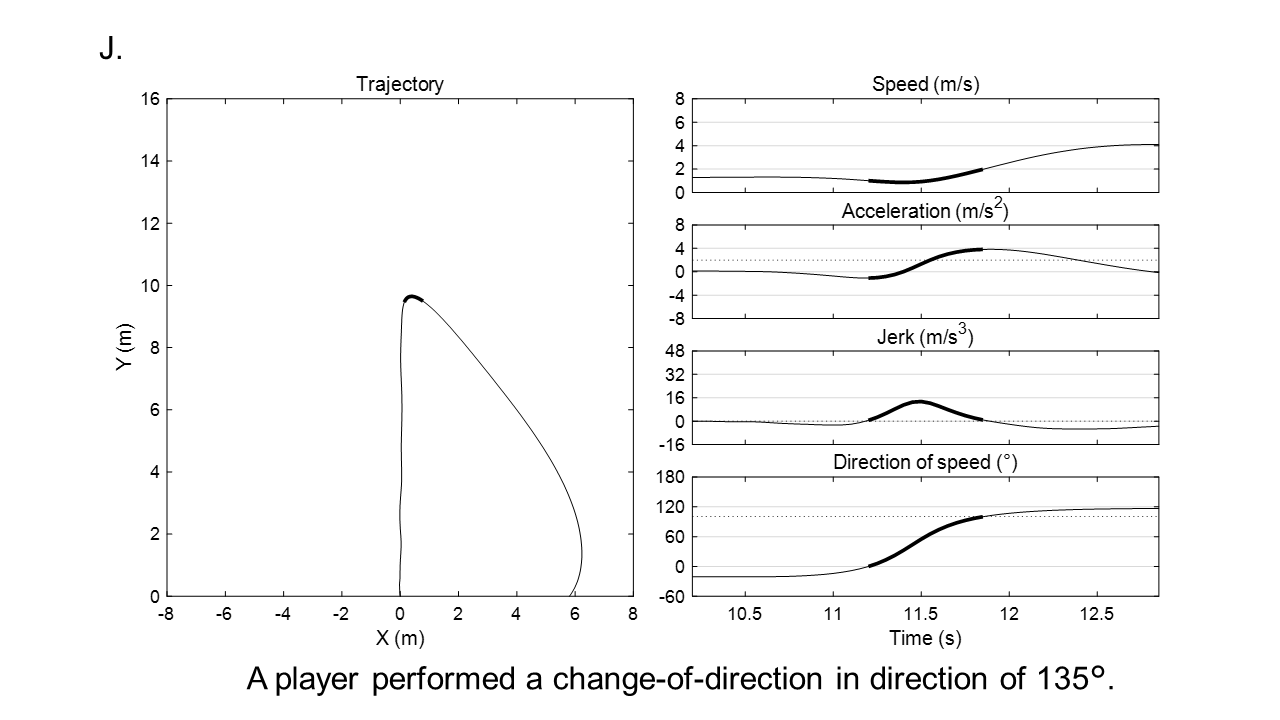

Supplement: S1 Fig — A: 0°, B: 15°, C: 30°, D: 45°, E: 60°, F: 75°, G: 90°, H: 105°, I: 120°, J: 135°, K: 150°, L: 165°, M: 180°. A bold line overlapped in line plot indicates an analytical period. (ZIP) [file pone.0251292.s001.zip › S1j_Fig.TIF]

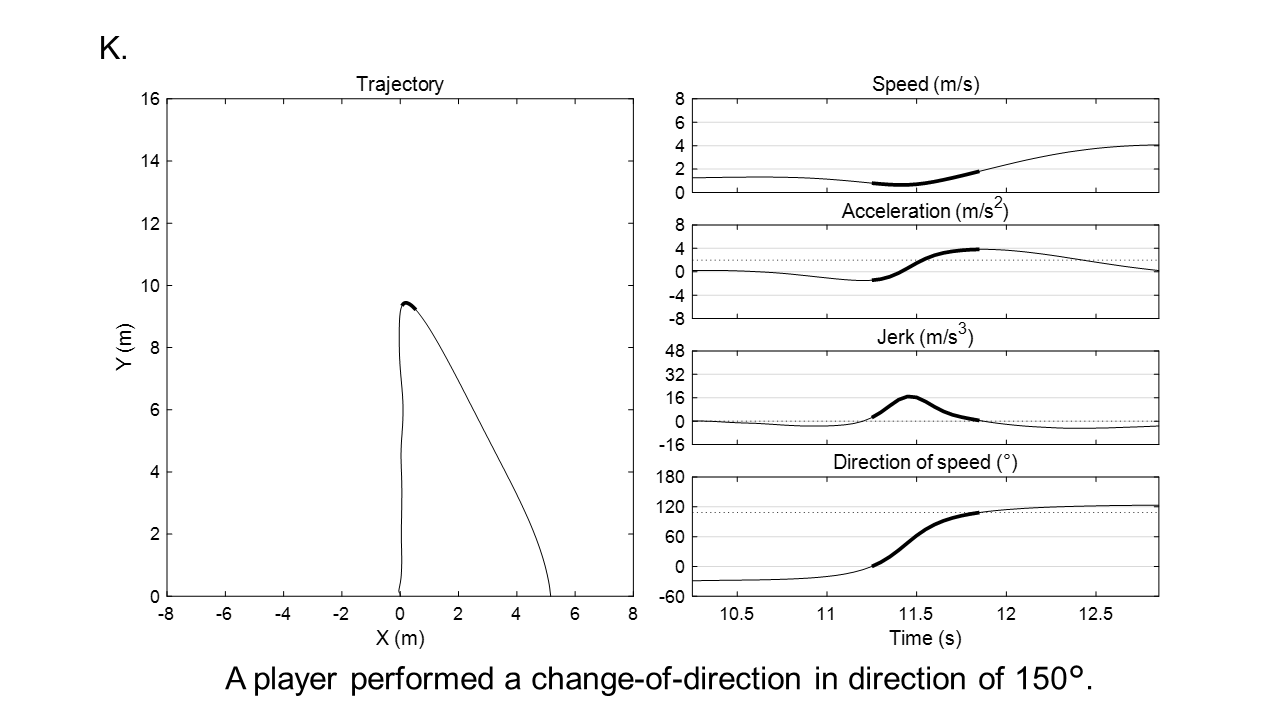

Supplement: S1 Fig — A: 0°, B: 15°, C: 30°, D: 45°, E: 60°, F: 75°, G: 90°, H: 105°, I: 120°, J: 135°, K: 150°, L: 165°, M: 180°. A bold line overlapped in line plot indicates an analytical period. (ZIP) [file pone.0251292.s001.zip › S1k_Fig.TIF]

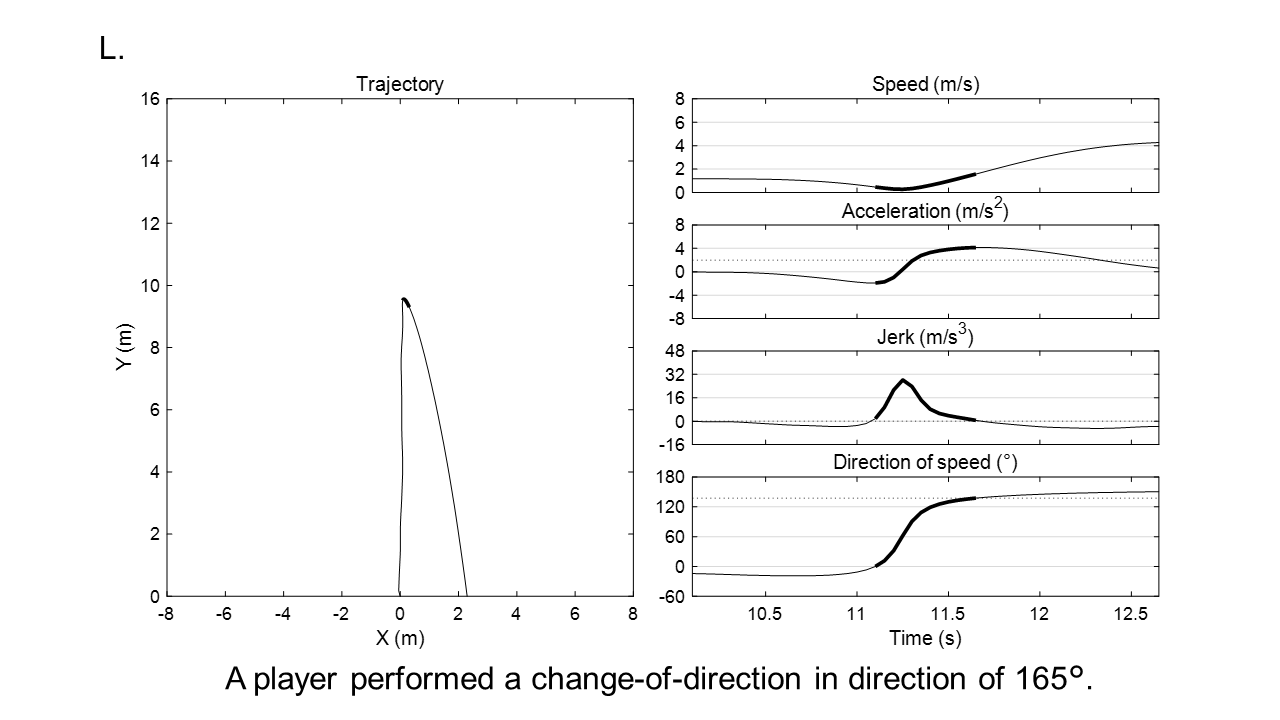

Supplement: S1 Fig — A: 0°, B: 15°, C: 30°, D: 45°, E: 60°, F: 75°, G: 90°, H: 105°, I: 120°, J: 135°, K: 150°, L: 165°, M: 180°. A bold line overlapped in line plot indicates an analytical period. (ZIP) [file pone.0251292.s001.zip › S1l_Fig.TIF]

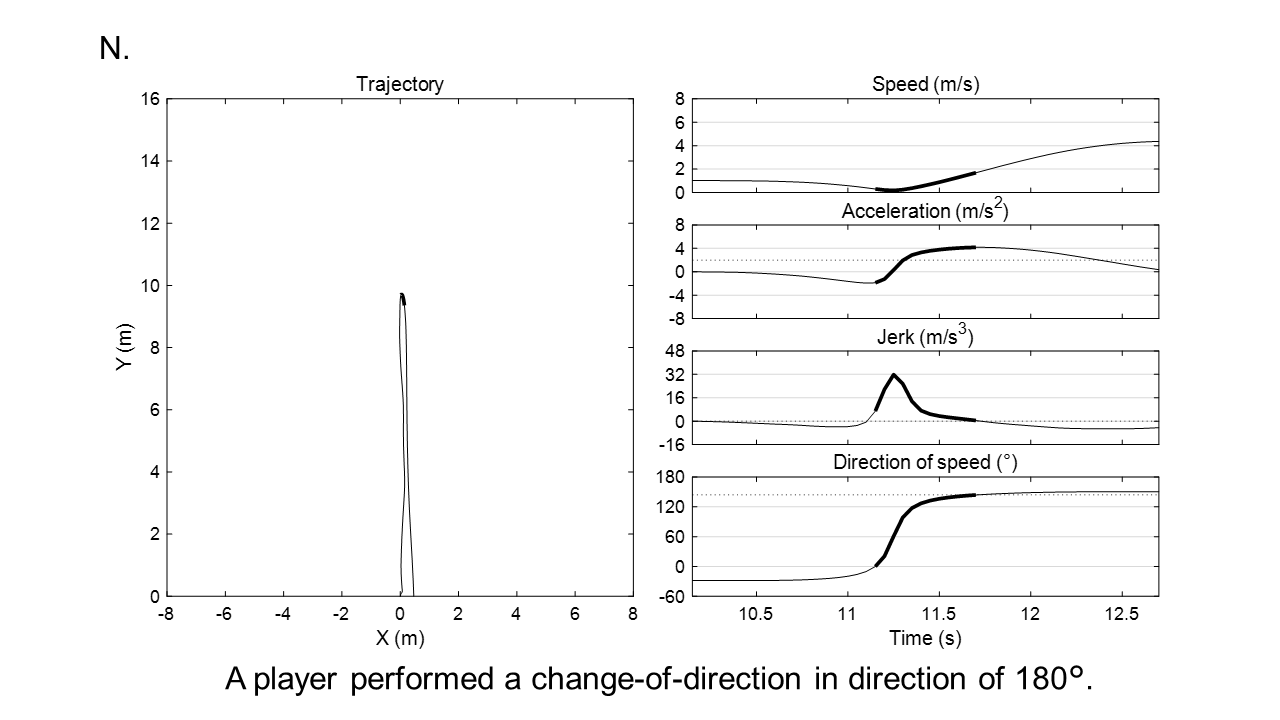

Supplement: S1 Fig — A: 0°, B: 15°, C: 30°, D: 45°, E: 60°, F: 75°, G: 90°, H: 105°, I: 120°, J: 135°, K: 150°, L: 165°, M: 180°. A bold line overlapped in line plot indicates an analytical period. (ZIP) [file pone.0251292.s001.zip › S1m_Fig.TIF]

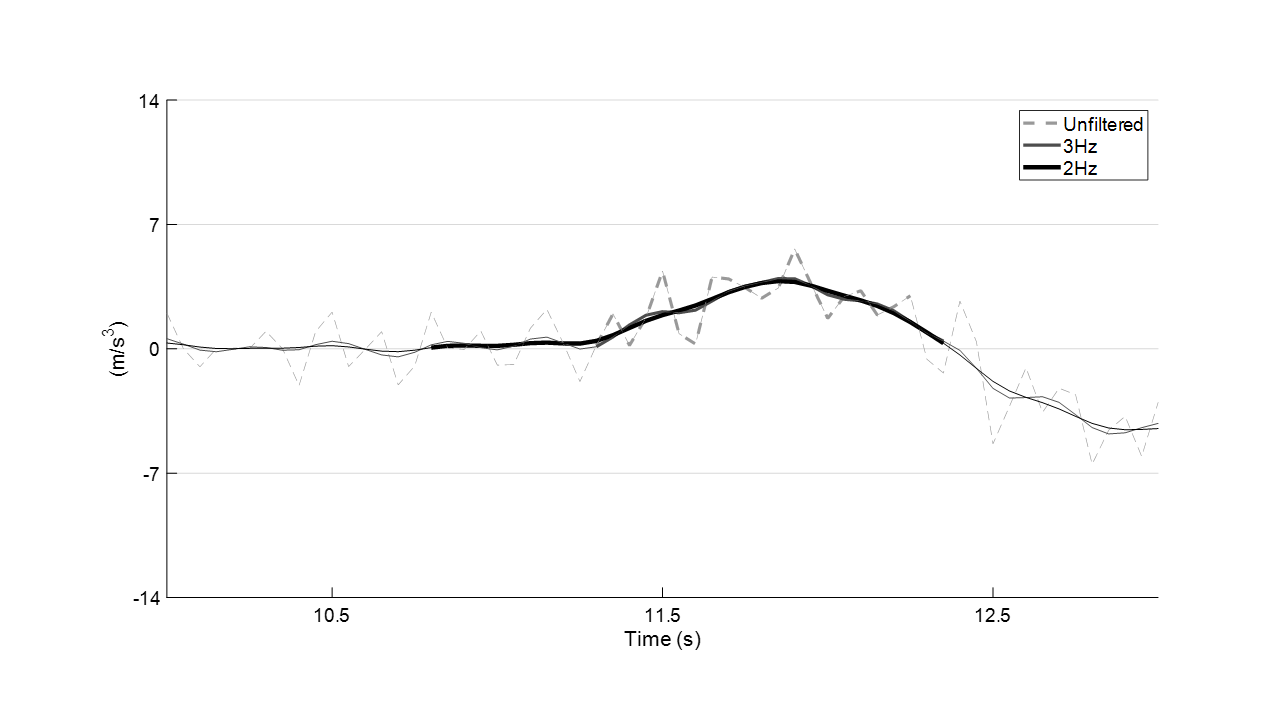

Supplement: S2 Fig — The use of cutoff frequencies of 3–6 Hz resulted in noise in the smoothed time-series data, while cutoff frequencies <2 Hz produced less noise. Thus, a 2 Hz cutoff frequency was adopted. (TIF) [file pone.0251292.s002.tif]
