# Supplementary material for: A new approach to quantify angles and time of changes-of-direction during soccer matches
Source: PLoS One. 2021 May 17;16(5):e0251292. doi: 10.1371/journal.pone.0251292 (PMC8128237; doi:10.1371/journal.pone.0251292)
Supplement: Attachment — Submitted filename: Reviewer document.pdf [file pone.0251292.s004.pdf]

Dear authors and editor,

It is a pleasure the opportunity to act as a reviewer for PLOS ONE. So, thank you very much for it. I have written my comments into two sections, the general comments with the main three ideas, and specific comments. With them, I hope to help the authors to improve the manuscript, encouraging them to perform due to the relevance of this topic.

General comments

- The lack of a deep literature review makes that introduction and discussion are overviews, but not further contextualization of "state of the art" or discussion of this topic.
- I am worried with the sampling rate considered. In sport literature, two systematic reviews have recently highlighted that tracking technologies are limited in high intensity short efforts such as CODs [1,2]. However, the authors considered 20 Hz for data raw and then they down sampling. Are these sampling rates suitable for this analysis? or is it a limitation?
- In my opinion, the results section shows a deep analysis, that does not meet with the rest of the article. The results showed:

1. The difference between COD and set angle.
2. Number and duration of COD during matches.
3. Number of CODs per playing position.

Regarding to the results 1 and 2, the aim should be: the validity of a new approach to set angles and duration of CODs. These aims are contextualized and fit with the rest of the sections (introduction and discussion), however, the reason to present data per playing position is not contextualized. Why do the authors consider it?

Specific comments

Title:

- Re-consider changing "locomotion" by "set angles and time of CODs".

Abstract:

- "CODs" is not defined the first time in it was mentioned.
- The "background" of abstract not contextualize the main objective. It should be something like: "Soccer players frequently change of directions (CODs) at various speeds during matches". However, tracking systems have shown limitations to measure these efforts. Therefore, the aim of the present study was to propose a new approach to measure CODs using a local positioning system (LPS)."

Introduction:

- The introduction lacks from relevant literature, making non suitable the contextualization of the objective. I suggest:

    - Paragraph 1: Explain the importance of change of directions. Maybe, different studies performed using PCA are suitable option to highlight the importance of change of directions and other high-intensity short efforts [3]. (The idea has been written, but in my opinion, further information is needed).
    - Paragraph 2: It should explain:
        - What are the tracking technologies (the main basis to use the new proposal that the authors presented).
        - What it has been found about tracking systems and change of directions.
        - What has it been the main problem of accuracy in this regard (e.g sampling rate).
    - Paragraph 3: it should explain "the state of the art" about all studies published in this topic (LPS and change of direction). Consider, at least, these references: [4–8]. Following this recently publicized systematic review [1], these all the validity proposals using LPS in sport setting.

*In general, the introduction has interesting ideas, but it should be deeply rewritten.

Methodology:

Separate "participants" from "experimental design".

Participants:

Add:

- Where do the players selected? What inclusion/exclusion criteria were considered?
- Participants characteristics.
- See any scale for risk of bias, and follow it.

Experimental design:

- Line 77-78: CODs in directions determined by <u>13</u> set angles. My question is: 3 or 13 set angles?

Players´ coordinate data:

- Sampling frequency: the authors mentioned 20 Hz (and less with filtering processed). However, the use of 20 Hz has not been enough for high intensity short efforts using LPS. Why do the authors think that in this study could be suitable sampling frequency? Could be a limitation? What was the criteria to use these sampling rates?
- The authors mentioned that an LPS was used, but, several factors could affect the outcome that they could not be related with the proposed approach. Therefore, a higher precision about "the use of technology" should be made,

mainly to avoid the following principle: “A good experimental design is one in which the only explanation for the change in the dependent variable is due to the treatment applied”. I suggest the use of recently published survey [9].

Results

The results are well-conducted, but they have different ways ((i) the difference between COD and set angle, (ii) number and duration of COD during matches, and (iii) number of CODs per playing position)) that are not reflected in the rest of the article. In my opinion, the article should be adapted to performed results (see general comments).

Consider explain the three protocols in the abstract.

Discussion:

- Re-write the discussion, considering the suggested articles for the introduction (paragraph 3).
- Reconsider separate a discussion for each of the results presented: (i) the difference between COD and set angle, (ii) number and duration of COD during matches, and (iii) number of CODs per playing position. If all of them are relevant for the aim of this study (see general comments).
- Add limitations.

Conclusion:

See the comments mentioned above. If following the results the aim was different folds, the conclusions should have different folds too.

References:

A further revision of the literature is needed. Different articles have been published about the use of LPS to assess CODs, and some of them were not considered.

Bibliography:

1. Rico-González M, Arcos AL, Clemente FM, Rojas-Valverde D, Pino-Ortega J. Accuracy and Reliability of Local Positioning Systems for Measuring Sport Movement Patterns in Stadium-Scale: A Systematic Review. Applied Sciences. 2020;10:5994.

2. Pino-Ortega J, Oliva-Lozano JM, Gantois P, Nakamura FY, Rico-González M. Comparison of the validity and reliability of local positioning systems against other tracking technologies in team sport: A systematic review. Proc IMechE Part P: J Sports Engineering and Technology. 2021;

3. Oliva-Lozano JM, Rojas-Valverde D, Gómez-Carmona CD, Fortes V, Pino-Ortega J. Impact of contextual variables on the representative external load profile of Spanish professional soccer match-play: A full season study. European Journal of Sport Science. 2020;1–10.

4. Frencken WGP, Lemmink KAPM, Delleman NJ. Soccer-specific accuracy and validity of the local position measurement (LPM) system. Journal of Science and Medicine in Sport. 2010;13:641–5.

5. Ogris G, Leser R, Horsak B, Kornfeind P, Heller M, Baca A. Accuracy of the LPM tracking system considering dynamic position changes. Journal of Sports Sciences. 2012;30:1503–11.

6. Stevens TGA, de Ruiter CJ, van Niel C, van de Rhee R, Beek PJ, Savelsbergh GJP. Measuring Acceleration and Deceleration in Soccer-Specific Movements Using a Local Position Measurement (LPM) System. International Journal of Sports Physiology and Performance. 2014;9:446–56.

7. Linke D, Link D, Lames M. Validation of electronic performance and tracking systems EPTS under field conditions. Ardigò LP, editor. PLOS ONE. 2018;13:e0199519.

8. Luteberget LS, Spencer M, Gilgien M. Validity of the Catapult ClearSky T6 Local Positioning System for Team Sports Specific Drills, in Indoor Conditions. Front Physiol. 2018;9:115.

9. Rico-González M, Arcos AL, Rojas-Valverde D, Clemente FM, Pino-Ortega J. A Survey to Assess the Quality of the Data Obtained by Radio-Frequency Technologies and Microelectromechanical Systems to Measure External Workload and Collective Behavior Variables in Team Sports. Sensors. 2020;16.
