# Supplementary material for: A new approach to quantify angles and time of changes-of-direction during soccer matches
Source: PLoS One. 2021 May 17;16(5):e0251292. doi: 10.1371/journal.pone.0251292 (PMC8128237; doi:10.1371/journal.pone.0251292)
Supplement: Attachment — Submitted filename: PLOSONE_ResponseToComment_v1.docx [file pone.0251292.s005.docx]

**Response to Reviewer #1 comments**

We thank the referees for fruitful suggestions, especially for suggesting the better context of introduction and discussion in this study. We have revised the manuscript on the basis of the referee's comments. We look forward to a publication of our manuscript in PLoS One.

**[General comments]**

**Comment #1**

The lack of a deep literature review makes that introduction and discussion are overviews, but not further contextualization of “state of the art” or discussion of this topic.

**Response to the comment**

We have agreed. We have revised description of the introduction and discussion in the revised text, according to the following comments. Please refer to the following responses to the corresponding comments.

**Comment #2**

I am worried with the sampling rate considered. In sport literature, two systematic reviews have recently highlighted that tracking technologies are limited in high intensity short efforts such as CODs [1,2]. However, the authors considered 20 Hz for data raw and then they down sampling. Are these sampling rates suitable for this analysis? or is it a limitation?

**Response to the comment**

The coordinate data was not decimated. We have analyzed the data after filter processing. As the reviewer comments, tracking technologies are limited in high intensity short effort. We have considered one of the limitations in this point. So, we have added the description of limitation to the revised text.

“There are some limitations in this study. Firstly, LPS are limited in high intensity effort such as high speed straight running and CODs [3, 4, 6, 9]. During sport-specific course and small-sided games, the root mean square errors of instant speed over 4.17 m/s range from 0.34 m/s to 0.39 m/s [4]. However, the values may not be enough to change relationships between instant speed at onset of CODs and angles of CODs (Fig. 4).”

**Comment #3**

In my opinion, the results section shows a deep analysis, that does not meet with the rest of the article. The results showed:

1. The difference between COD and set angle.

2. Number and duration of COD during matches.

3. Number of CODs per playing position.

Regarding to the results 1 and 2, the aim should be: the validity of a new approach to set angles and duration of CODs. These aims are contextualized and fit with the rest of the sections (introduction and discussion), however, the reason to present data per playing position is not contextualized. Why do the authors consider it?

**Response to the comment**

We have added the description of position-related difference in CODs to the revised text. According to both reviewers’ comment, we have revised context of the introduction and discussion mainly. Please refer to the revised text.

**[Specific comments]**

**Comment #4**

Title: Re-consider changing “locomotion” by “set angles and time of CODs”.
**Response to the comment**

We have agreed. We have changed “changes-of direction” by “angles and time of changes-of-direction” in the revise text.

**Comment #5**

Abstract: “CODs” is not defined the first time in it was mentioned.

**Response to the comment**

We have defined change-of-directions as the term of CODs in the revised text.

**Comment #6**

Abstract: The “background” of abstract not contextualize the main objective. It should be something like: “Soccer players frequently change of directions (CODs) at various speeds during matches”. However, tracking systems have shown limitations to measure these efforts. Therefore, the aim of the present study was to propose a new approach to measure CODs using a local positioning system (LPS).”

**Response to the comment**

According to the reviewer’s comment, we have done.

**Comment #7**

The introduction lacks from relevant literature, making non suitable the contextualization of the objective. I suggest:

Paragraph 1: Explain the importance of change of directions. Maybe, different studies performed using PCA are suitable option to highlight the importance of change of directions and other high-intensity short efforts [3]. (The idea has been written, but in my opinion, further information is needed).

Paragraph 2: It should explain:
What are the tracking technologies (the main basis to use the new proposal that the authors presented).
What it has been found about tracking systems and change of directions.

What has it been the main problem of accuracy in this regard (e.g sampling rate).

Paragraph 3: it should explain “the state of the art” about all studies published in this topic (LPS and change of direction). Consider, at least, these references: [4–8]. Following this recently publicized systematic review [1], these all the validity proposals using LPS in sport setting.

*In general, the introduction has interesting ideas, but it should be deeply rewritten.

**Response to the comment**

We have agreed. According to both reviewer’s comments, we have revised description of the introduction section as follows;

“Recently, coaches and sports scientists of ball sports team use tracking technologies for designing training program and players’ condition, because it has become easy to measure players’ locations in ball sports using global positioning system (GPS) and local positioning system (LPS) [1, 2]. Many earlier studies have demonstrated the validity of position, speed and acceleration data obtained from such a tracking technology, compared to a 3D motion capture system, radar/laser guns and timing gates [3-9]. Further, LPS is superior to GPS, whereas each system has a certain validity [10]. These systems are capable of measuring players’ coordinates, and for quantifying players’ acceleration, distance and numbers of actions in relation to speed derived from time-motion analysis. These parameters are predictors of match outcome and periodization on daily training [11, 12].

Besides time-motion analysis, the parameters obtained from tracking technologies are applied to quantify CODs locomotion in sport-specific course and small-sided games [3-6, 9, 10]. In the earlier studies, various type of courses which angles of CODs are predetermined are set [3, 5, 6, 9, 10], and small court is used [4]. Although the earlier findings demonstrate the magnitude of speed and acceleration, the experimental design is not real soccer matches. During soccer matches, players perform changes-of-direction (CODs) during locomotion [13]. Notational analysis has revealed that 30% of all actions during English FA Premier League play were CODs (e.g., forward, lateral and backward running) [14]. However, quantifying relevant data, such as the number and type of actions, is a lengthy process [15], and notational analysis may be arbitrary [16]. Therefore, a convenient analytic method to quantify CODs during soccer matches is needed. Fitzpatrick et al. [2] demonstrated that, in the English U-18 Premier League, the direction of players’ locomotion at a speed of 6.67 m/s or more ranged from 0° to 30° by using GPS. This suggests that during matches, youth soccer players move close to a straight line at relatively high speeds. Further, an earlier study of soccer matches revealed that greater distances are covered at moderate speeds of 3.89 to 5.28 m/s than at high-intensity speeds of 5.28 to 6.39 m/s [17]. Although soccer players frequently perform CODs at various speeds during matches, to the best of our knowledge, little information is available concerning COD profiles during soccer matches in relation to speed by using LPS.

Force is theoretically the product of mass and acceleration. Acceleration can be useful in describing a player’s physical load during soccer matches. Dalen et al. [1] demonstrated that position-related differences in the number of accelerations (>2 m/s^2^) was found in the first division of the Norwegian league. When a player changes direction of locomotion, he exerts a certain force against the ground. At the same time, a certain level of acceleration is produced, and then the direction of the player’s locomotion changes, and the speed and/or the direction of speed changes [3]. Jerk, which is derived by differentiating acceleration by time, is used to detect the onset of human joint movement and the magnitude of the movement [18]. Therefore, jerk should be useful in identifying the onset and end of a COD for a given acceleration, and the change in direction of speed should correspond to the direction of the COD.

During professional soccer matches, position-related differences in acceleration are found, indicating that side midfielders and defenders accelerate more often than other positions [1]. Further, midfielders, relative to other positions, performed fewer CODs of 90 degrees or less [14]. Considering these findings, profile of CODs would differ among positions. This study thus proposes a new approach that uses direction of speed and jerk in quantifying CODs of soccer players during soccer matches, and to clarify position-related difference in profile of CODs by using the approach.”

**Methodology**

**Comment #8**

Separate “participants” from “experimental design”.

**Response to the comment**

We have done. Please refer to the revised text.

**Comment #9**

Participants:

Add:

Where do the players selected? What inclusion/exclusion criteria were considered?

Participants characteristics. See any scale for risk of bias, and follow it.

**Response to the comment**

We have added the corresponding description to the revised text.

“In the Exp. 1, six collegiate male soccer players (age, 21.0 ± 1.5 years, height, 172.8 ± 6.1 cm, body mass, 66.8 ± 9.2 kg; means ± SDs) participated in Exp. 1. They were field players, and belonged in the same team competing in a national university league in Japan, and had experience of competitive soccer training for >9 years. They had participated in regular soccer-specific training for more than five days (>1.5 hours/day) per week.

Seventy-nine collegiate and amateur male soccer players (23.0 ± 4.1 years, 173.9 ± 5.1 cm, 67.5 ± 6.2 kg) involved in Exp. 2, and got in the official soccer matches in in Division 1 of a regional collegiate male soccer league or the Division 5 of a regional amateur soccer league. The number of players in each position was as follows; 23 players for central backs (CB, 23.4 ± 4.7 years, 177.4 ± 5.0 cm, 71.2 ± 5.7 kg), 16 players for side backs (SB, 22.6 ± 3.4 years, 171.7 ± 3.9 cm, 65.2 ± 4.7 kg), 15 players for central midfielders (CMF, 22.2 ± 2.9 years, 172.3 ± 5.0 cm, 64.8 ± 6.3 kg), 14 players for side midfielders (SMF, 23.7 ± 5.0 years, 172.3 ± 4.2 cm, 65.9 ± 4.9 kg), and 11 players for forwards (FW, 21.8 ± 3.7 years, 174.3 ± 4.5 cm, 69.8 ± 5.9 kg), respectively. Goalkeepers were excluded from data analysis.

All participants involved in Exp. 1 and 2 were free of cardiovascular, metabolic, and immunologic disorders and/or orthopedic abnormalities, and were not using any medications that affected their muscular function and size.”

**Comment #10**

Experimental design:

Line 77-78: CODs in directions determined by 13 set angles. My question is: 3 or 13 set angles?

**Response to the comment**

In this study, we have set 13 angles (every 15°, ranging from 0° to 180°).

**Comment #11**

Players´ coordinate data:

Sampling frequency: the authors mentioned 20 Hz (and less with filtering processed). However, the use of 20 Hz has not been enough for high intensity short efforts using LPS. Why do the authors think that in this study could be suitable sampling frequency? Could be a limitation? What was the criteria to use these sampling rates?

**Response to the comment**

As the reviewer comments, the use of 20 Hz has not been enough for high intensity efforts using LPS. Unfortunately, the system used in this study is only capable for measuring at 20 Hz when more than 10 players wear the sensors. Hence, we used sampling rate of 20 Hz. It could be one of the limitations, and we have added the description to discussion section in the revised text.

“There are some limitations in this study. Firstly, LPS are limited in high intensity effort such as high speed straight running and CODs [3, 4, 6, 9]. During sport-specific course and small-sided games, the root mean square errors of instant speed over 4.17 m/s range from 0.34 m/s to 0.39 m/s [4]. However, the values may not be enough to change relationships between instant speed at onset of CODs and angles of CODs (Fig. 4).”

**Comment #12**

The authors mentioned that an LPS was used, but, several factors could affect the outcome that they could not be related with the proposed approach. Therefore, a higher precision about “the use of technology” should be made, mainly to avoid the following principle: “A good experimental design is one in which the only explanation for the change in the dependent variable is due to the treatment applied”. I suggest the use of recently published survey [9].

**Response to the comment**

We have agreed. The earlier studies have demonstrated the validity of tracking technology (GPS and LPS) concerning position, speed and acceleration data compared to a 3D motion capture system, radar/laser guns and timing gates cited by #3-#9. In this study, the course predetermined by 13 set angles was set to quantify CODs (Exp. 1). As the results, *θ*_COD_ increased with increasing set angles. The current result indicates that angle of CODs can be estimated by jerk above a certain acceleration. On the other hand, we have considered the reviewer’s point is important to interpret our data. Unfortunately, we have the relevant data, so we have added a limitation of this point to the revised text.

“Thirdly, parameters derived from LPS may be influenced by measurement condition and experimental protocol [26], although the validity of tracking systems is shown in earlier studies abovementioned [3-9]. Unfortunately, we have the relevant data, and further investigation is needed in this point.”

**Comment #13**

Results

The results are well-conducted, but they have different ways ((i) the difference between COD and set angle, (ii) number and duration of COD during matches, and (iii) number of CODs per playing position)) that are not reflected in the rest of the article. In my opinion, the article should be adapted to performed results (see general comments).
**Response to the comment**

We have agreed. We have added description of position-related difference in CODs during soccer matches to the revised text. Please refer to response to comment #7

**Comment #14**

Consider explain the three protocols in the abstract.

**Response to the comment**

We have done.

Discussion:

**Comment #15**

Re-write the discussion, considering the suggested articles for the introduction (paragraph 3).

**Response to the comment**

We have agreed. With changing description of the introduction (see response to comment #7), we have revised content of the discussion in the corresponding part.

“The mean values of analytical duration were 1.04 s for Exp. 1, and 0.89 s for Exp. 2. Granero-Gil et al. [23] have defined change-of-direction locomotion as curvilinear locomotion that lasts more than 0.8 s, and they attempted to detect CODs during soccer matches by using an inertial sensor. The analytical durations in the present study were close to this definition, providing support for the threshold reported by Granero-Gil et al. [23]. On the other hand, the number of CODs in the present matches was approx. 183, lower than the corresponding value (471 times) reported by Granero-Gil et al. [23]. This discrepancy may be due to differences in the method of analysis. In this study, *θ*_COD_ was estimated based on acceleration above 2 m/s^2^ [1], while the earlier study used the definition above to identify changing-of-direction locomotion [23]. Another reason may be due to be the technical error of acceleration measured by LPS. In fact, Linke et al. [4] have demonstrated that root mean square error of acceleration in sport-specific course ranged from 0.49 m/s^2^ to 1.34 m/s^2^.”

**Comment #16**

Reconsider separate a discussion for each of the results presented: (i) the difference between COD and set angle, (ii) number and duration of COD during matches, and (iii) number of CODs per playing position. If all of them are relevant for the aim of this study (see general comments).

**Response to the comment**

We have agreed. With changing the context of the introduction, according to both reviewers’ comments, we have revised the contents of the discussion. Please refer to the revised text.

**Comment #17**

Add limitations.

**Response to the comment**

We have done.

“There are some limitations in this study. Firstly, LPS are limited in high intensity effort such as high speed straight running and CODs [3, 4, 6, 9]. During sport-specific course and small-sided games, the root mean square errors of instant speed over 4.17 m/s range from 0.34 m/s to 0.39 m/s [4]. However, the values may not be enough to change relationships between instant speed at onset of CODs and angles of CODs (Fig. 4). Secondly, the approach used in this study cannot be used to determine direction of a body. For example, if a player runs backward in the opposite direction immediately after he moves in a straight run, the locomotion is estimated as a COD with a 180° turn. Further investigation of this point is needed. Thirdly, parameters derived from LPS may be influenced by measurement condition and experimental protocol [26], although the validity of tracking systems is shown in earlier studies abovementioned [3-9]. Unfortunately, we have the relevant data, and further investigation is needed in this point.”

Conclusion:

**Comment #18**

See the comments mentioned above. If following the results the aim was different folds, the conclusions should have different folds too.
**Response to the comment**

We have changed the corresponding part as follows;

“This study proposed a new approach to quantifying angle of CODs (*θ*_COD_) during soccer matches by using direction of speed and jerk. As the results, *θ*_COD_ increases with increasing the predetermined set angle, although *θ*_COD_ was smaller than the predetermined set angle. Further, the relative frequency of *θ*_COD_ derived from the proposed approach revealed position-specific, and *θ*_COD_ was affected by speed at the onset of the COD during soccer matches. The current findings suggest that the approach proposed in this study may be useful to quantify angle of CODs during soccer matches.”

**Comment #19**

References:

A further revision of the literature is needed. Different articles have been published about the use of LPS to assess CODs, and some of them were not considered.
**Response to the comment**

We have appreciated for introducing the relevant references. We have added the relevant references to the revised text.

**Response to Reviewer #2 comments**

We thank the referees for fruitful suggestions, especially for suggesting the better context of introduction and discussion. We have revised the manuscript on the basis of the referee's comments. We look forward to a publication of our manuscript in PLoS One.

Abstract

**Comment #1**

L25. Please, include information about participants.

**Response to the comment**

We have changed the following description in the revised text.

“Six collegiate male soccer players performed CODs according to 13 set angles (0-180°; every 15°) so that differences between *θ*_COD_ and set angle could be determined (Exp. 1). Relative frequency distributions of *θ*_COD_ and number of CODs were determined in 79 collegiate and amateur male soccer players during 9 soccer matches (Exp. 2)”

**Comment #2**

In general, I expect a more structured abstract. Please, follow this order and include the presented information in the section that corresponds to it:

- Background and aim

- Methods

- Results

- Conclusions

**Response to the comment**

We have done.

Introduction

**Comment #3**

The first paragraph must be improved. Although it shows the necessity of perform this study, the previous justification is poor, in quality and quantity.

**Response to the comment**

We have agreed. We have revised content of introduction section as follows;

“Recently, coaches and sports scientists of ball sports team use tracking technologies for designing training program and players’ condition, because it has become easy to measure players’ locations in ball sports using global positioning system (GPS) and local positioning system (LPS) [1, 2]. Many earlier studies have demonstrated the validity of position, speed and acceleration data obtained from such a tracking technology, compared to a 3D motion capture system, radar/laser guns and timing gates [3-9]. Further, LPS is superior to GPS, whereas each system has a certain validity [10]. These systems are capable of measuring players’ coordinates, and for quantifying players’ acceleration, distance and numbers of actions in relation to speed derived from time-motion analysis. These parameters are predictors of match outcome and periodization on daily training [11, 12].”

**Comment #4**

In the second paragraph, more information about local positioning systems and its relationship with COD before to show examples. In addition, this paragraph must be improved in terms of style.

L48. Please, include the complete terms for GPS and LPS.

L57. Reference.

L 61. Reference.

L62. Reference.

**Response to the comment**

We have agreed. We have added the following sentences to the corresponding part in the revised text.

“Recently, coaches and sports scientists of ball sports team use tracking technologies for designing training program and players’ condition, because it has become easy to measure players’ locations in ball sports using global positioning system (GPS) and local positioning system (LPS) [1, 2]. Many earlier studies have demonstrated the validity of position, speed and acceleration data obtained from such a tracking technology, compared to a 3D motion capture system, radar/laser guns and timing gates [3-9]. Further, LPS is superior to GPS, whereas each system has a certain validity [10]. These systems are capable of measuring players’ coordinates, and for quantifying players’ acceleration, distance and numbers of actions in relation to speed derived from time-motion analysis. These parameters are predictors of match outcome and periodization on daily training [11, 12]. ”

**Comment #5**

Please, divide the third paragraph and include a fourth one, highlighting the necessity of perform this investigation, the main aim, and the hypothesis.

**Response to the comment**

We have agreed. We have changed as follows;

“Force is theoretically the product of mass and acceleration. Acceleration can be useful in describing a player’s physical load during soccer matches. Dalen et al. [1] demonstrated that position-related differences in the number of accelerations (>2 m/s^2^) was found in the first division of the Norwegian league. When a player changes direction of locomotion, he exerts a certain force against the ground. At the same time, a certain level of acceleration is produced, and then the direction of the player’s locomotion changes, and the speed and/or the direction of speed changes [3]. Jerk, which is derived by differentiating acceleration by time, is used to detect the onset of human joint movement and the magnitude of the movement [18]. Therefore, jerk should be useful in identifying the onset and end of a COD for a given acceleration, and the change in direction of speed should correspond to the direction of the COD.

During professional soccer matches, position-related differences in acceleration are found, indicating that side midfielders and defenders accelerate more often than other positions [1]. Further, midfielders, relative to other positions, performed fewer CODs of 90 degrees or less [14]. Considering these findings, profile of CODs would differ among positions. This study thus proposes a new approach that uses direction of speed and jerk in quantifying CODs of soccer players during soccer matches, and to clarify position-related difference in profile of CODs by using the approach.”

Material and methods

**Comment #6**

Participants and experimental design

This section must be divided, on one hand experimental design, an in the other hand, participants. In addition, information must be structured (following a logical order) and complete. In this sense, more information about participants is required.

**Response to the comment**

We have done.

“*Experimental design”*

This investigation was conducted according to the Declaration of Helsinki and approved by the local Ethics Committee for human experimentation (#11-101). All participants were informed of the experimental procedures and possible risks of the measurements beforehand. Oral informed consent was obtained from each subject before each match.

This study consisted of two experiments for quantifying CODs during soccer matches in relation to various speeds. In both experiments, players’ locations were measured with LPS. In the first experiment 1 (Exp. 1), male soccer players performed CODs in directions determined by 13 set angles (every 15°, ranging from 0° to 180°) while jogging at speeds of approximately 1.0 and 3.0 m/s. After the player turned at the determined location, he ran through a gate set at a distance of 2 m from the corresponding location. The participants were asked to perform CODs as fast and quickly as possible when they turned in a given direction. S1 Fig. presents typical trajectory data and kinematic data (to be discussed below) in each angle for one player.

In the second experiment 2 (Exp. 2), data were collected from 9 official soccer matches in Division 1 of a regional collegiate male soccer league and the Division 5 of a regional amateur soccer league for collegiate and amateur soccer players. Data were analyzed for the players who played for 90 min.

***Participants***

In the Exp. 1, six collegiate male soccer players (age, 21.0 ± 1.5 years, height, 172.8 ± 6.1 cm, body mass, 66.8 ± 9.2 kg; means ± SDs) participated in Exp. 1. They were field players, and belonged in the same team competing in a national university league in Japan, and had experience of competitive soccer training for >9 years. They had participated in regular soccer-specific training for more than five days (>1.5 hours/day) per week.

Seventy-nine collegiate and amateur male soccer players (23.0 ± 4.1 years, 173.9 ± 5.1 cm, 67.5 ± 6.2 kg) involved in Exp. 2, and got in the official soccer matches in in Division 1 of a regional collegiate male soccer league or the Division 5 of a regional amateur soccer league. The number of players in each position was as follows; 23 players for central backs (CB, 23.4 ± 4.7 years, 177.4 ± 5.0 cm, 71.2 ± 5.7 kg), 16 players for side backs (SB, 22.6 ± 3.4 years, 171.7 ± 3.9 cm, 65.2 ± 4.7 kg), 15 players for central midfielders (CMF, 22.2 ± 2.9 years, 172.3 ± 5.0 cm, 64.8 ± 6.3 kg), 14 players for side midfielders (SMF, 23.7 ± 5.0 years, 172.3 ± 4.2 cm, 65.9 ± 4.9 kg), and 11 players for forwards (FW, 21.8 ± 3.7 years, 174.3 ± 4.5 cm, 69.8 ± 5.9 kg), respectively. Goalkeepers were excluded from data analysis.

All participants involved in Exp. 1 and 2 were free of cardiovascular, metabolic, and immunologic disorders and/or orthopedic abnormalities, and were not using any medications that affected their muscular function and size.”

**Comment #7**

Players’ coordinate data

I consider that this section is ok, but an effort by authors to facilitate its compression is required. In addition, the use of the first person style must be avoided.

**Response to the comment**

We have agreed. We have changed description of the first person style to that of passive form style. Further, we have added our effort to facilitate to the revised text as follows;

“X- and y-coordinate data for each player were measured with LPS (ZXY Sports Tracking, Chyronhego, New York, USA) at a sampling frequency of 20 Hz. A belt with a sensor (approx. 20 g) under their uniform were attached for each player. In Exp.2, three examiners helped to wear the sensor for starters before the start of soccer matches. Players were instructed to take off the belt if they felt uncomfortable during matches. The data obtained were processed as described below using Matlab (Mathworks ver. 2018b, New York, USA).”

**Comment #8**

Statistical analysis

More information for this section is required.

**Response to the comment**

We have done.

**Comment #9**

Results

Complete information is presented in this section, although improvements in style must be implemented.

**Response to the comment**

We have done.

**Comment #10**

Discussion

Please, for the first paragraph follow this structure: Aim – novelty of the study – main findings.

This section must be improved completely. More information about justifications (with references) and comparisons with previous studies is required.

**Response to the comment**

We have done.

“This study aims to propose a new approach that uses direction of speed and jerk in quantifying CODs of soccer players during soccer matches, and to clarify position-related difference in profile of CODs by using the approach. As the results, change in direction of speed, *θ*_COD_, which was derived from direction of speed and jerk, increased with increasing set angles of the predetermined course (Exp. 1). Further, the relative frequency of *θ*_COD_ during soccer matches revealed a platykurtic distribution in positions other than FW, but, in that of FW, the distribution was leptokurtic (Exp. 2). Therefore, the *θ*_COD_ proposed in this study may be an index of angle of CODs, and profile of CODs during soccer matches obtained from *θ*_COD_ may be position-specific.”

**Comment #11**

Please, include a limitation section.

**Response to the comment**

We have agreed. We have added the limitation of this study to the revised text.

“There are some limitations in this study. Firstly, LPS are limited in high intensity effort such as high speed straight running and CODs [3, 4, 6, 9]. During sport-specific course and small-sided games, the root mean square errors of instant speed over 4.17 m/s range from 0.34 m/s to 0.39 m/s [4]. However, the values may not be enough to change relationships between instant speed at onset of CODs and angles of CODs (Fig. 4). Secondly, the approach used in this study cannot be used to determine direction of a body. For example, if a player runs backward in the opposite direction immediately after he moves in a straight run, the locomotion is estimated as a COD with a 180° turn. Further investigation of this point is needed. Thirdly, parameters derived from LPS may be influenced by measurement condition and experimental protocol [26], although the validity of tracking systems is shown in earlier studies abovementioned [3-9]. Unfortunately, we have the relevant data, and further investigation is needed in this point.”

**Comment #12**

Conclusion

Please, only show the most relevant conclusions derivated of your findings, avoiding first person style.

**Response to the comment**

We have changed the corresponding part as follows;

“This study proposed a new approach to quantifying angle of CODs (*θ*_COD_) during soccer matches by using direction of speed and jerk. As the results, *θ*_COD_ increases with increasing the predetermined set angle, although *θ*_COD_ was smaller than the predetermined set angle. Further, the relative frequency of *θ*_COD_ derived from the proposed approach revealed position-specific, and *θ*_COD_ was affected by speed at the onset of the COD during soccer matches. The current findings suggest that the approach proposed in this study may be useful to quantify angle of CODs during soccer matches.”

**Comment #13**

Please, include practical applications derivated of yours finding that can be used by strength and conditioning specialists.

**Response to the comment**

We have agreed. We have added the following sentences to the revised text.

“In practical application, this study demonstrated that the relative distribution of *θ*_COD_ was position-specific, and *θ*_COD_ was affected by speed at the onset of the COD during soccer matches. To design regular soccer training that meet physical demands for each position, coaches and strength conditioners for soccer players have to know what kind of locomotion is taking place during soccer matches. Considering the current findings, the players of positions other than FW need to perform CODs toward various direction at relatively low speed. For FW, however, it’s better to perform CODs toward narrow angle at relatively high speed. Thus, the current findings may be useful information to achieve the principle of training specificity for soccer.”
